# The Impact of Vaping on the Ocular Surface: A Systematic Review of the Literature

**DOI:** 10.3390/jcm13092619

**Published:** 2024-04-29

**Authors:** Nilanga Aki Bandara, Barbara Burgos-Blasco, Xuan Randy Zhou, Amar Khaira, Alfonso Iovieno, Joanne A. Matsubara, Sonia N. Yeung

**Affiliations:** 1 UBC Faculty of Medicine, Department of Medicine, Vancouver, BC V6T 1Z3, Canada; 2 UBC Faculty of Medicine, Department of Ophthalmology and Visual Sciences, Vancouver, BC V5Z 3N9, Canada; barbara.burgos@salud.madrid.org (B.B.-B.);; 3 Vagelos School of Physicians & Surgeons, Columbia University, New York City, NY 10027, USA; xz3042@cumc.columbia.edu

**Keywords:** vaping, ocular surface, electronic cigarette, cornea, eye

## Abstract

**Background:** The use of electronic cigarettes has become increasingly popular in recent years. However, the impact that electronic cigarettes have on the ocular surface is not well known. Therefore, the aim of this review is to explore the current literature on the acute and chronic sequelae of electronic cigarettes on the ocular surface. **Methods:** A systematic review of the literature was undertaken by keyword searching on the Embase, Medline, and Web of Science databases. Articles identified through the search underwent title/abstract screening, full-text screening, and data extraction. **Results:** A total of 18 studies were included in this review. Non-intended ocular surface exposures and intended exposures on the ocular surface were found to be associated with the use of electronic cigarettes. **Conclusions:** The impact of vaping on the ocular surface is not benign. There are significant risks that vaping can pose to the ocular surface. Hence, it is necessary to develop appropriate risk communication tools given the increasing popularity of this activity. Additionally, future long-term studies are needed to better understand the long-term impacts of vaping on the ocular surface given the lack of current data.

## 1. Introduction

Electronic cigarette (e-cig) and vaping products have increased in popularity in recent years. Globally, it has been estimated that roughly 68 million people used e-cigs in the year 2020 [1], highlighting the widespread use of these products. E-cigs and vaping products have been discussed as an alternative to combustible cigarettes [2]. Some proponents of e-cigs and vaping products suggest that these products may even have a role as a potential harm reduction tool [3]. Qualifying these products as a harm reduction tool suggests that the use of e-cigs or vaping products may be safer than the use of combustible cigarettes. In fact, some users of combustible cigarettes are also dual users of e-cigs and vaping products, highlighting that users may use these different products, instead of using a single product [3]. E-cigs and vaping products often come in various designs and typically contain a battery, heater, and e-liquid [2]. Some e-cig users may even consider modifying their e-cigs through a variety of mechanisms such as by changing their e-cig battery power or coil resistance [4]. Modifying these products may put users at risk of unintended outcomes.

There exists a great deal of controversy surrounding e-cigs, vaping products, and their safety, some of which has been amplified by misinformation on social media platforms [5,6]. During the COVID-19 pandemic, one instance of misinformation highlighted the potential for e-cigs to be protective against the COVID-19 virus [5]. This spread of misinformation on social media may have impacted and influenced vulnerable populations in our society, such as youth, to use e-cigs in an effort to protect themselves from COVID-19 [5]. Unfortunately, the e-cig or vaping-associated lung injury (EVALI) outbreak has shown some of the dangers that can be associated with the use of e-cigs, such as acute respiratory distress syndrome or acute eosinophilic pneumonia [6]. Further, it is known that e-cigs contain nicotine, which is a highly addictive substance [7]. Addiction to nicotine is one of the major barriers that can make smoking cessation efforts challenging, therefore highlighting one of the main challenges of e-cigs or vaping products as a smoking cessation tool [7]. The development of optimal risk communication tools to engage people who use e-cigs is needed [5], so that they can understand the potential risks associated with e-cig use and make informed decisions about using these products.

Nicotine and other chemicals in e-cigs may affect different components of the eye including the corneal epithelium, the tear film, and retinal light-adapted vision, along with inducing nystagmus and exerting vasoconstrictive effects on the ocular blood flow [8]. The ocular surface includes the surface and glandular epithelia of the cornea, conjunctiva, lacrimal gland, accessory lacrimal glands, and meibomian gland, and their apical (tears) and basal (connective tissue) matrices, the eyelashes with their associated glands of Moll and Zeis, those components of the eyelids responsible for the blink, and the nasolacrimal duct and its health are critical for providing and maintaining clear vision [9,10]. Currently, there exists a paucity of data on the impact that e-cig and vaping product use have on the ocular surface [8,11]. In a review by Martheswaran et al. [8], two contrasting perspectives on the effects that e-cigs and vaping have on the ocular surface were described [12,13]. Thus, it is still unclear what the true impact of e-cigs or vaping products is on the ocular surface, highlighting the need for further exploration and study. Given the detrimental effects that e-cigs or vaping products have on other physiological systems in the body [5,6] and the nearby proximity of the ocular surface to e-cig or vaping product exposure, the main aim of this review was to specifically explore the impact of e-cig or vaping product use on the ocular surface.

## 2. Materials and Methods

The research question this systematic review aimed to answer was, What impact does e-cig or vaping product use have on the ocular surface? A formal search strategy was developed and finalized with the assistance of a subject-expert librarian at the University of British Columbia. The systematic review was not registered. A primary literature search was performed using the Embase, Medline, and Web of Science databases with keywords and MeSH terms including “e-cig*” or “e cig*” or “electronic cig*” or “vaping and ocular surface or cornea* or conjunctiv*”. MeSH terms were identified and used as appropriate to strengthen the search. After carrying out the initial search, articles of interest were uploaded onto the research management software Covidence (covidence.org, Veritas Health Innovation Ltd.; Melbourne, Australia) [14]. After uploading onto the Covidence software, duplicates were automatically identified and removed by Covidence. The authors manually checked each article automatically flagged as a duplicate to ensure that articles were not inaccurately being removed. The patient, exposure, and outcome (PEO) framework was used to establish the inclusion and exclusion criteria for this review.

### 2.1. Inclusion Criteria

For the population, human participants of any age, geographic location, or gender identity were included. For exposure, participants had to have some degree of exposure to e-cigs or vaping products. The primary outcome of interest was the impact that e-cig or vaping use has on the ocular surface. All study designs were deemed to be eligible for this systematic review, including original research and review articles. Moreover, articles from any publication date and only peer-reviewed publications were included.

### 2.2. Exclusion Criteria

Studies without any discussion of research on human participants, for example, studies that were focused on animal models, were excluded. Studies where the exposure did not include data on e-cigs or vaping products—in other words, studies that only focused on traditional, combustible cigarette use—as well as studies where there were no outcomes related to the ocular surface, for example, studies that focused on the retina or other ocular structures, were also excluded. Articles that were not available in the English language were excluded, as the authors did not have the resources to reliably and accurately translate non-English research studies. In addition, non-peer-reviewed materials, such as publications in the grey literature (blogs, interviews, and dissertations), were excluded from the present review.

Following this, each article underwent title and abstract screening against the review’s inclusion and exclusion criteria. Eligible articles then underwent full-text screening where an article’s full text was evaluated against the review’s inclusion and exclusion criteria. Finally, articles that were deemed to be eligible went through data extraction. At each stage of the review, two independent reviewers (NAB and XRZ) assessed the eligibility of every article. If there were any conflicts between the reviewers, the two reviewers (NAB and XRZ) met, discussed the article, and came to consensus. While no issues of missing full-text articles or retrieving specific articles were faced, had such issues arisen, a plan to identify the corresponding author, contact them, and request for access to their paper had been created.

**Figure 1:**
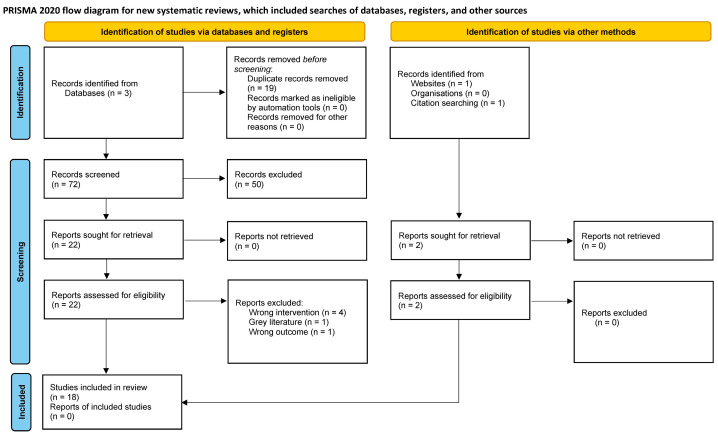
PRISMA flowchart.

The secondary literature was found using relevant references from the articles identified by the primary literature search and through a search of the Google Scholar website. Following the primary literature search on the three formal databases, 16 articles were identified. Further, after the secondary search, an additional two articles were identified. Therefore, 18 articles were identified, reviewed, and included in this systematic review. These 18 articles were then thematically analyzed to be coded under non-intended exposures to the ocular surface or intended exposure to the ocular surface. Exposures where research participants were unexpectedly exposed to the contents of e-cigs or vaping products were classified as non-intended exposures. On the other hand, in intended exposures, users were aware of their e-cig use. Figure 1 shows the Preferred Reporting Items for Systematic Reviews and Meta-Analyses (PRISMA) [15] flowchart for this review, which documents all steps of this systematic review from study identification to data extraction.

## 3. Results

After data extraction, the diverse effects that e-cigs had on the ocular surface through non-intended exposures and intended (acute and chronic) exposures were conceptualized.

### 3.1. Non-Intended

Non-intended exposures are exposures caused by the explosions of e-cigs or vaping products, inadvertent exposure to e-liquid, and exposures related to components of e-cigs or vaping products breaking off. These types of exposures are not typically expected with routine use of e-cigs or vaping products, but may occur accidentally [16,17,18,19].

Spontaneous e-cig device explosions may cause unintended injuries to e-cig users; these explosions are difficult to predict and therefore to avoid. Documented injuries from e-cig explosions include corneal injury (epithelial defects and corneal burn), subconjunctival hemorrhage, and black particulate accumulation in the tear film and conjunctiva [16,17,18,19]. The severity of these injuries can vary significantly. Patterson et al. [18] described a patient with an abrasion of 80% of the cornea from an e-cig explosion. Another case report presented an explosion injury due to a modified e-cig involving the right side of his face that initially required irrigation of the ocular surface due to inadvertent contact with alkaline fluid [20]. The patient presented with two 2cm linear superficial upper eyelid laceration wounds that did not involve the eyelid margin and an 8mm conjunctival laceration, both of which required suturing. In addition, there was traumatic mydriasis, anterior uveitis and cataract with zonulysis, extensive commotio retinae, and Berlin oedema.

Accidental exposure to e-liquid is another cause for concern. Several case reports have highlighted a multitude of possible ways one could be exposed to the chemicals and nicotine in e-cigs, mainly through accidental ingestion and ocular exposure [21,22,23,24]. There are also reports of accidental e-liquid ocular application when mistaken for eye drops; the ocular surface damage in these cases ranged from mild eye irritation to ocular chemical injury [22,23,24]. These inadvertent exposures to e-liquid differ in severity and often lead to poison centre calls that require additional allocation of health resources [25].

These non-intended ocular exposures are significant and constitute the majority of poison control centre calls in the United States. Wang et al. [26] found that the majority of the calls (87%) had a root exposure that was ocular only, further underlining the importance of communicating these risks to care providers.

### 3.2. Intended Exposures

Intended exposures point to the potential short-term (acute) and long-term (chronic) effects that e-cigs or vaping products have on the ocular surface.

### 3.3. Acute Exposure

Munsamy et al. [13] assessed the impact that acute exposure to e-cig use had on the ocular surface. Specifically, they assessed tear film stability and corneal epithelial thickness before and following acute exposure to e-cigs, at a dose equivalent to about 10 puffs in 64 participants. Non-invasive tear breakup time (NITBUT) is a useful indicator of the stability of the tear film [27]. An increase in NITBUT of 1.4 s after e-cig use was found, but this was not statistically significant (*p* = 0.089), which could be due to the limited time between e-cig use and NITBUT measurement, as well as the variability of instrumentation [13]. Also, the chosen number of puffs (0.05 mL of e-liquid or on average 10 puffs of e-cigarette vapour) may have been inadequate to effect changes. After e-cig use, there was an increase in the mean corneal epithelial thickness in all five zones (central, superior, inferior, nasal, and temporal), although this was also not statistically significant (*p* > 0.05) [13].

### 3.4. Chronic Exposure

Ocular surface malignancies, changes in tear film stability and quality, tear production, corneal epithelial thickness, average loss of meibomian glands in the upper and lower eyelid, and dry eye symptoms/ocular irritation have been associated with chronic exposure to e-cig use [11,28,29]. An earlier comprehensive review carried out by Makrynioti et al. [11] described the impact that various forms of smoking, such as combustible cigarettes and e-cigs, had on dry eye and various ocular conditions. Their work suggests that while research on e-cigs is still in its infancy, there are some signs that point to e-cigs resulting in ocular irritation and dry eye disease.

Shields et al.’s [28] case report highlighted that e-cig use may be associated with the development of conjunctival intraepithelial neoplasia (CIN). The patient had used e-cigs for five years at a rate of at least five vapes per day without any concurrent cigarette use. The authors hypothesized a correlation between the carcinogenic components of e-cigs and the development of CIN.

Another chronic effect is the disruption of the ocular surface components such as the tear film quality and stability and the meibomian glands’ physiology. Dry eye or eye irritation can be caused by disruption of the normal functioning of the tear film and meibomian glands [30]. In a cross-sectional study, Md Isa et al. [12] found that there was a significant reduction in the NITBUT of e-cig users compared to a control group (non-e-cig users) over a one-year period [12]. Moreover, a systematic review conducted by Miglio et al. [31] revealed that e-cigs could negatively impact both the stability and quality of the tear film. Kalayci et al. [29] conducted a cross-sectional study comparing the ocular surface of people who used and did not use e-cigs (control). Tear breakup time (TBUT) was evaluated with the assistance of a biomicroscope, and e-cig users had a significantly lower TBUT of 6.96 ± 2.31 s compared to 9.84 ± 2.13 s for the control group [29].

As for tear production, Md Isa et al. [12] used the Schirmer test with topical anaesthesia to evaluate this in long-term e-cig users compared to controls. Tear production amongst those in the e-cig group was significantly higher at an average of 14.5 mm compared to an average of 8.0 mm for the control group (*p* = 0.001). This increase in tear production may be a potential homeostatic measure in response to lower tear film integrity that may result from e-cig use. In contrast, Kalayci et al. [29] found that the average Schirmer II values were significantly (*p* = 0.002) lower in the e-cig group at 9.16 ± 2.09 mm compared to the control group at 11.2 mm ± 2.14 mm, which was attributed to the impact that the metabolites of lipid peroxidation (i.e., malondialdehyde) associated with e-cig use have on the ocular surface.

Furthermore, Kalayci et al. [29] conducted non-contact meibography to evaluate the impact of e-cig use on the meibomian glands over an average duration of 4.9 years. The loss of meibomian glands was calculated using the Phoenix meibography imaging software module installed on the Sirius (CSO, Florence, Italy) corneal topography device. After taking images of the tarsal conjunctiva, the borders of the eyelids and of the meibomian glands are marked using the Phoenix software module. The meibomian gland loss is automatically calculated by the software, obtaining values of the percentage loss. Their study found that participants in the e-cig group had a significant (*p* = 0.002) average meibomian gland loss of 23.08 ± 6.55% on the upper eyelid and a significant (*p* < 0.001) average meibomian gland loss of 27.83 ± 5.98% on the lower eyelid compared to the controls who had an average loss of 17.60 ± 4.94% and 18.44 ± 5.91%, respectively. Moreover, e-cig users were more likely to have Meibomian glands that were irregularly distributed and less hyperreflective compared to the control group [29].

Finally, Md Isa et al. [12] used the Ocular Surface Disease Index (OSDI) score to assess dry eye symptoms in participants who reported long-term use of e-cigs compared to controls; their study noted that participants in the e-cig group had moderate to severe dry eye syndrome compared to those in the control group [12]. Also, Kalayci et al. [29] observed that the OSDI score was significantly higher in the e-cig group compared to the control group (*p* < 0.001). Interestingly, dry eye symptoms were shown to substantially increase in relation to an increase in e-cig voltage [12]. Thus, participants who used e-cigs with greater voltage were more likely to experience more severe dry eye syndrome [12].

### 3.5. Quality Assessment

In general, the research studies included in this systematic review highlight that the quality of evidence available on how the use of e-cigs and vaping products impact the ocular surface is low. None of the research studies included in this review were randomized control trials (RCTs). It is necessary to acknowledge that much of the research on how e-cigs impact the ocular surface is likely still emerging, which may potentially explain why the majority of the studies included in this systematic review (68%) were in the form of case reports and case series. Also, many of the case series were often limited to small sample sizes, which further reduces the generalizability of their findings.

## 4. Discussion

This review highlights that e-cig use is associated with both non-intended and intended exposure adverse effects on the ocular surface. Published studies demonstrated that e-cigs can have a negative impact on the stability and quality of the tear film, which is an important component of ocular surface health. This can lead to an increase in dry eye syndrome in users of e-cigs. Therefore, the impact of e-cigs or vaping products on the ocular surface is not benign.

The non-intended impacts that e-cigs can have on the ocular surface point out potential dangers that e-cig users may not be aware of. For example, e-cig explosions may result in ocular trauma, such as subconjunctival hemorrhages [16] or corneal abrasions [16,17,18,19,20]. Based on this review, corneal trauma was the most common form of ocular surface injury as a result of e-cig explosions. There are a variety of reasons why e-cigs explode, including, in particular, modifying the different components of e-cigs [20,32]. Further, Jamison and Lockington [23] and Hughes and Hendrickson [24] described how e-liquid containers can be mistaken for eye drops, particularly as the containers can appear similar. Since most calls to poison control centers concern ocular-related injuries, with almost 20% of these injuries pertaining to children under the age of five [26], clear communication regarding the risks of e-cig use is clearly needed. Efforts to educate e-cig users on the ocular risks that can be associated with e-cig modifications may help prevent associated injuries. Clear labeling and possibly the use of very distinct containers for e-liquid may also help in preventing inadvertent administration to the ocular surface. Making warning messages mandatory and highly visible on e-liquids may also potentially reduce inadvertent exposure of the ocular surface to e-liquids.

A variety of acute and chronic impacts on the ocular surface have been described. The potential carcinogenic effects of long-term e-cig use were highlighted by Shields et al. [30]. Given the fact that research on the possible oncologic outcomes associated with e-cig and vaping use is still emerging, users of these products should be made aware of the potential risks of ocular-related oncological conditions in order to make an informed decision on the use of these products. Further, several groups investigated the impact that e-cig use had on the stability of the tear film and dry eye syndrome/ocular irritation and together yielded conflicting results [12,13,14]. This could be due to the very low dose used in Munsamy et al.’s [13] study, which did not show an impact of e-cig use on the ocular surface. Participants used e-cigs at a dose equivalent to that of about 10 puffs, while typical e-cig users may inhale anywhere from 120 to 235 puffs per day [33]. On the other hand, the participants in Md Isa et al.’s [12] and Kalayci et al.’s [29] studies may be more representative of typical e-cig users given their average daily e-cig use frequency and duration of e-cig use.

An interesting point for future analysis is the impact that e-cigs have on tear production. Md Isa et al. [12] observed that e-cig use was associated with increased tear production, while Kalayci et al. [29] noted that e-cig use led to decreased tear production. Considering the duration of e-cig use, Md Isa et al.’s [12] sample had been using e-cigs for an average of 17.2 ± 6.5 months, while Kalayci et al.’s [29] sample had an average use of 4.9 ± 0.9 years. Therefore, long-term use of e-cigs may be associated with a reduced level of tear production. Exploring this further, the reduced amount of tear production could be due to use over the long term in combination with the effects of various aldehydes, which have the potential to cause lipid peroxidation, thereby damaging the tear film’s lipid layer and impacting the stability of the tear film. This could lead to a long-term reduction in tear production [34].

As this area of study is still unraveling, reviews are limited by the type of studies and level of evidence available to date. For instance, 68% of studies were either case reports or small case series, which underscores the overall low quality of data available on this topic. Miglio et al.’s [31] article was the only systematic review included in this review, which mainly addressed the work of Md Isa et al. [12]. Additionally, our systematic review did not include research studies from the grey literature and sources from outside our primary and secondary literature search; therefore, additional perspectives and research identified by these sources may not have been included in this review. Although more work certainly needs to be carried out to determine with more certainty the long-term impacts of e-cig usage on the ocular surface, there is low-level evidence to date to suggest the negative impact on various components of the ocular surface, with particular reference to the development of dry eye syndrome. However, it is important to acknowledge that an absence of evidence of harm at this stage does not mean that long-term use of e-cigs is safe.

## 5. Conclusions

This review showcases both the non-intended and intended impact that e-cigs can have on the ocular surface. Primarily, e-cigs may have a negative impact on the ocular surface by reducing the quality and stability of the tear film. Since the prevalence of dry eye disease can be significant in the general population, it is important to recognize e-cigs and vaping products as a potential contributing factor to this multifactorial disease entity. As the use of e-cigs and vaping products continues to increase in popularity, considering this factor in the evaluation and management of dry eye disease will become more important.

Given these early findings, efforts should be made to educate e-cig users on the potential effects that vaping can have on their ocular surface, as well as the potential dangers of non-intended exposures. Additionally, policymakers should consider implementing legislation that reduces the potential for ocular harm caused by e-cigs or vaping products, such as restrictions on the containers that e-liquids are available in. Future research should include larger cross-sectional studies and aim at better characterizing the long-term impact that e-cig use has on the ocular surface, especially considering that research on e-cigs and the ocular surface is still emerging. A risk analysis of such effects among a variety of e-cig users, such as daily versus occasional users, may also be of interest to better qualify the relationship between frequency of e-cig use and relevant health outcomes. Further, comparative studies analyzing the impact on the ocular surface of both combustible cigarettes and e-cigs will allow us to better understand whether a true role exists for e-cigs as a harm reduction tool. Future studies should also consider including dual users of e-cigs or vaping products and cigarettes, as that can allow for a comparison of outcomes between only e-cig users, only combustible cigarette users, and dual users.

## References

[1] Jerzyński T., Stimson G.V., Shapiro H., Król G. (2021). Estimation of the global number of e-cigarette users in 2020. Harm Reduct. J..

[2] Breland A., Soule E., Lopez A., Ramôa C., El-Hellani A., Eissenberg T. (2017). Electronic cigarettes: What are they and what do they do?. Ann. N. Y. Acad. Sci..

[3] Berg C.J., Haardoerfer R., Escoffery C., Zheng P., Kegler M. (2015). Cigarette Users’ Interest in Using or Switching to Electronic Nicotine Delivery Systems for Smokeless Tobacco for Harm Reduction, Cessation, or Novelty: A Cross-Sectional Survey of US Adults. Nicotine Tob. Res..

[4] Mulder H.A., Patterson J.L., Halquist M.S., Kosmider L., Turner J.B.M., Poklis J.L., Poklis A., Peace M.R. (2019). The Effect of Electronic Cigarette User Modifications and E-liquid Adulteration on the Particle Size Profile of an Aerosolized Product. Sci. Rep..

[5] Bandara N.A., Herath J., Mehrnoush V. (2021). Addressing e-cigarette health claims made on social media amidst the COVID-19 pandemic. World J. Pediatr..

[6] Bandara N.A., Jhauj R., Fernando J., Mehrnoush V., Wijesinghe N. (2021). Overlapping public health crises during the coronavirus disease pandemic. World J. Emerg. Med..

[7] Bandara N.A., Seneviratne M., Wanniarachchi S., Mehrnoush V. (2020). E-cigarettes and youth health. Br. Columbia Med. J..

[8] Martheswaran T., Shmunes M.H., Ronquillo Y.C., Moshirfar M. (2021). The impact of vaping on ocular health: A literature review. Int. Ophthalmol..

[9] Gipson I.K. (2007). The Ocular Surface: The Challenge to Enable and Protect Vision: The Friedenwald Lecture. Investig. Ophthalmol. Vis. Sci..

[10] Shumway C.L., Motlagh M., Wade M. (2022). Anatomy, Head and Neck, Eye Conjunctiva.

[11] Makrynioti D., Zagoriti Z., Koutsojannis C., Morgan P.B., Lagoumintzis G. (2020). Ocular conditions and dry eye due to traditional and new forms of smoking: A review. Contact Lens Anterior Eye.

[12] Md Isa N.A., Koh P.Y., Doraj P. (2019). The Tear Function in Electronic Cigarette Smokers. Optom. Vis. Sci. Off. Publ. Am. Acad. Optom..

[13] Munsamy A., Bhanprakash B., Sirkhot A., Mlambo L., Dlamuka S., Mhlongo N., Naidoo R. (2019). A pre-test post-test assessment of non-invasive keratograph break up time and corneal epithelial thickness after vaping. Afr. Health Sci..

[14] *Covidence Systematic Review Software*; Covidence: Melbourne, Australia. https://www.covidence.org/.

[15] PRISMA. http://prisma-statement.org/prismastatement/flowdiagram.aspx.

[16] Paley G.L., Echalier E., Eck T.W., Hong A.R., Farooq A.V., Gregory D.G., Lubniewski A.J. (2016). Corneoscleral Laceration and Ocular Burns Caused by Electronic Cigarette Explosions. Cornea.

[17] Cason D.E., Morgan D.E., Pietryga J.A. (2016). Injuries from an exploding e-cigarette: A case report. Ann. Intern. Med..

[18] Patterson S.B., Beckett A.R., Lintner A., Leahey C., Greer A., Brevard S.B., Simmons J.D., Kahn S.A. (2017). A Novel Classification System for Injuries after Electronic Cigarette Explosions. J. Burn Care Res. Off. Publ. Am. Burn Assoc..

[19] Anderson H., Richie C., Bernard A. (2017). A Surprisingly Volatile Smoking Alternative: Explosion and Burns as Risks of E-Cigarette Use. J. Burn Care Res..

[20] Khairudin M.N., Zahidin A.Z.M., Bastion M.-L.C. (2016). Front to back ocular injury from a vaping-related explosion. Case Rep..

[21] Gomolka E., Radomska M., Bielska D.E. (2016). Acute poisoning with e-cigarette liquid—Case report. Prz. Lek..

[22] Chatham-Stephens K., Law R., Taylor E., Kieszak S., Melstrom P., Bunnell R., Wang B., Day H., Apelberg B., Cantrell L. (2016). Exposure Calls to U. S. Poison Centers Involving Electronic Cigarettes and Conventional Cigarettes-September 2010–December 2014. J. Med. Toxicol..

[23] Jamison A., Lockington D. (2016). Ocular chemical injury secondary to electronic cigarette liquid misuse. JAMA Ophthalmol..

[24] Hughes A., Hendrickson R.G. (2018). Inadvertent ocular exposures secondary to e-liquid misuse. Clin. Toxicol..

[25] Wang B., Liu S., Persoskie A. (2020). Poisoning exposure cases involving e-cigarettes and e-liquid in the United States, 2010–2018. Clin. Toxicol..

[26] Wang B., Liu S.T., Johnson M.A., Trigger S. (2022). Trends and characteristics of ocular exposures related to e-cigarettes and e-liquids reported to Poison Control Centers in the United States, 2010–2019. Clin. Toxicol..

[27] Wolffsohn J.S., Arita R., Chalmers R., Djalilian A., Dogru M., Dumbleton K., Gupta P.K., Karpecki P., Lazreg S., Pult H. (2017). TFOS DEWS II Diagnostic Methodology report. Ocul. Surf..

[28] Shields C.L., Kim M., Lally S.E., Chevez-Barrios P., Shields J.A. (2020). Eye cancer in a young male with a vaping history. Indian J. Ophthalmol..

[29] Kalayci M., Cetinkaya E., Yaprak L., Yigit K., Suren E., Dogan B., Erol M.K. (2022). Ocular surface assessment and morphological alterations in meibomian glands with non-contact meibography in electronic cigarette smokers. Arq. Bras. Oftalmol..

[30] Bron A.J., de Paiva C.S., Chauhan S.K., Bonini S., Gabison E.E., Jain S., Knop E., Markoulli M., Ogawa Y., Perez V. (2017). TFOS DEWS II pathophysiology report. Ocul. Surf..

[31] Miglio F., Naroo S., Zeri F., Tavazzi S., Ponzini E. (2021). The effect of active smoking, passive smoking, and e-cigarettes on the tear film: An updated comprehensive review. Exp. Eye Res..

[32] Seitz C.M., Kabir Z. (2018). Burn injuries caused by e-cigarette explosions: A systematic review of published cases. Tob. Prev. Cessat..

[33] Etter J.-F., Eissenberg T. (2015). Dependence levels in users of electronic cigarettes, nicotine gums and tobacco cigarettes. Drug Alcohol Depend..

[34] Choi W., Lian C., Ying L., Kim G.E., You I.C., Park S.H., Yoon K.C. (2016). Expression of Lipid Peroxidation Markers in the Tear Film and Ocular Surface of Patients with Non-Sjogren Syndrome: Potential Biomarkers for Dry Eye Disease. Curr. Eye Res..

